# A Novel Nano-Antimicrobial Polymer Engineered with Chitosan Nanoparticles and Bioactive Peptides as Promising Food Biopreservative Effective against Foodborne Pathogen *E. coli O157*-Caused Epithelial Barrier Dysfunction and Inflammatory Responses

**DOI:** 10.3390/ijms222413580

**Published:** 2021-12-18

**Authors:** Ming Kuang, Haitao Yu, Shiyan Qiao, Tao Huang, Jiaqi Zhang, Mingchao Sun, Xiumei Shi, Han Chen

**Affiliations:** 1 Institute of Systems Biomedicine, Department of Immunology, School of Basic Medical Sciences, Beijing Key Laboratory of Tumor Systems Biology, Peking University Health Science Center, Beijing 100191, China; Kuang_ming@pku.edu.cn; 2 State Key Laboratory of Animal Nutrition, Ministry of Agriculture and Rural Affairs Feed Industry Center, College of Animal Science and Technology, China Agricultural University, Beijing 100193, China; qiaoshiyan@cau.edu.cn; 3 Beijing Key Laboratory of Urban Hydrological Cycle and Sponge City Technology, College of Water Sciences, Beijing Normal University, Beijing 100875, China; huangtao@bnu.edu.cn; 4 State Key Laboratory of Animal Nutrition, Institute of Animal Sciences of Chinese Academy of Agricultural Sciences, Beijing 100193, China; 82101191100@caas.cn (J.Z.); mingchao@stu.qau.ecu.cn (M.S.); 82101205353@caas.cn (X.S.); chenhan@stu.qau.edu.cn (H.C.)

**Keywords:** chitosan nanoparticles, antimicrobial peptide, microcin J25, foodborne pathogens, *E. coli O157*, anti-inflammation, intestinal epithelial barrier, nuclear factor κB, mitogen-activated protein kinase

## Abstract

For food quality and safety issues, the emergence of foodborne pathogenic bacteria has further accelerated the spread of antibiotic residues and drug resistance genes. To alleviate the harm caused by bacterial infections, it is necessary to seek novel antimicrobial agents as biopreservatives to prevent microbial spoilage. Nanoantimicrobials have been widely used in the direct treatment of bacterial infections. CNMs, formed by chitosan nanoparticles and peptides, are promising antibiotic alternatives for use as excellent new antibacterial drugs against pathogenic bacteria. Herein, the current study evaluated the function of CNMs in the protection of foodborne pathogen *Escherichia coli* (*E. coli*) O157 infection using an intestinal epithelial cell model. Antibacterial activity assays indicated that CNMs exerted excellent bactericidal activity against *E. coli O157*. Assessment of the cytotoxicity risks toward cells demonstrated that 0.0125–0.02% of CNMs did not cause toxicity, but 0.4% of CNMs caused cytotoxicity. Additionally, CNMs did not induced genotoxicity either. CNMs protected against *E. coli O157*-induced barrier dysfunction by increasing transepithelial electrical resistance, decreasing lactate dehydrogenase and promoting the protein expression of occludin. CNMs were further found to ameliorate inflammation via modulation of tumor factor α, toll-like receptor 4 and nuclear factor κB (NF-κB) expression via inhibition of mitogen-activated protein kinase and NF-κB activation and improved antioxidant activity. Taken together, CNMs could protect the host against *E. coli O157*-induced intestinal barrier damage and inflammation, showing that CNMs have great advantages and potential application as novel antimicrobial polymers in the food industry as food biopreservatives, bringing new hope for the treatment of bacterial infections.

## 1. Introduction

Despite the development and application of a large number of antibiotics, bacterial infections continue to pose a serious threat to human health; for instance, morbidities and mortality increases in intestinal inflammatory disease (IBD) induced by enteric diseases are universal problems [1,2,3,4]. Importantly, enterohemorrhagic *Escherichia coli* (EHEC) O157, a critical foodborne pathogen, infection is a major enteric pathogenic disease and primary cause of food contamination, diarrhea and death in humans, neonates and weaned animals. In addition, food spoilage caused by pathogenic microorganisms is also a cause of foodborne illness, which raises significant health concerns [5,6,7,8]. Antibiotic therapy has been used to control intestinal diseases for many years, but antibiotic resistance has rapidly emerged worldwide [9,10,11,12]. For severe drug-resistant bacterial infections, it is important to develop an effective treatment strategy. To meet the growing consumer demand for safe ready-to-eat foods, an attractive alternative to chemical preservatives is needed. Among these emerging approaches, nano-antimicrobials show great potential in the treatment of severe drug-resistant bacterial infections due to their excellent size effect, specific physicochemical properties and ease of modification [13].

In recent years, polymeric nanoparticles have included not only natural polymeric nanoparticles, such as those formed from antimicrobial peptides, but also synthetic polymeric nanoparticles [14,15,16]. The antimicrobial peptide microcin belongs to a class of bacteriocins [17,18,19] and chitosan micro/nanoparticles have facilitated attractive interest in using these molecules as new antibacterial alternatives to chemical preservatives or therapeutic agents [20,21,22,23]. In our previous study, we successfully designed and engineered CNMs, an antimicrobial nanohybrid that is composed of chitosan nanoparticles (CNs) and antimicrobial peptide (AMP) microcin J25 (MccJ25). CNMs could be an excellent novel antimicrobial agent against pathogen infection because of their excellent broad-spectrum antimicrobial activity without increasing the mutation rate. Moreover, the antimicrobial activity of CNMs remained stable in the face of different environmental conditions, improving their ease of storage and application. It is noteworthy that CNMs do not cause toxic risk [24]

In real*-world conditions, E. coli* can colonize intestinal epithelial cells, disrupt epithelial tight junction integrity and cause intestinal inflammation, ultimately inducing diarrhea and death [25,26,27,28]. EHEC infection is becoming a major public health concern [6,7,21,26,27,28]. Therefore, in this study, we investigate the safe, effective and nontoxic effects of CNMs as potential antimicrobials to protect against pathogen infections. Here, because intestinal epithelial cell (IPEC) J2 cells possess high resemblance between pigs and humans, they are a better model of normal intestinal epithelial cells for studying pathogen–host interactions and intestinal barrier function. Hence, we studied the protective capacity of CNMs against *E. coli* O157-induced intestinal barrier dysfunction and inflammatory responses using an IPEC-J2 cell model. 

## 2. Results

### 2.1. Effects of CNMs at Different Working Concentrations on The Cytotoxicity of IPEC-J2 Cells

**Figure 1:**
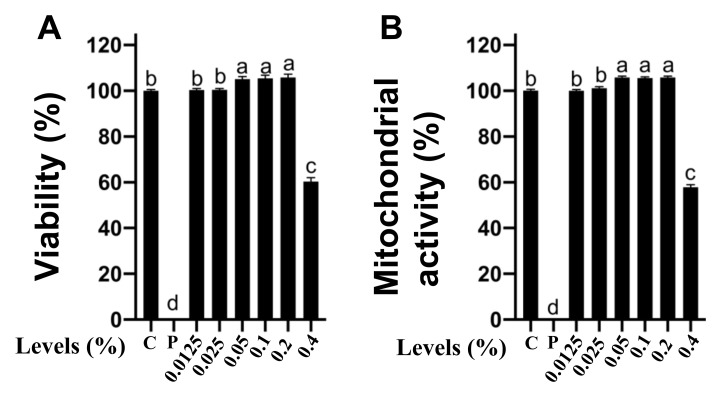
Cytotoxicity analysis of CNMs toward intestinal epithelial cells. LDH assay (**A**) and MTT assay (**B**) of IPEC-J2 cells in the presence of various concentrations of CNMs. Positive control was Triton X-100. Data are the means ± SEM of three independent experiments. Means with different lowercase letters differ significantly (*p* < 0.05). C, negative control; *p*, positive control.

It is critical to ensure that CNMs are not toxic to the host at antimicrobial concentrations prior to application to the host [29]. The first stage of the risk assessment was to test whether CNMs produced cytotoxicity to IPEC-J2 cells, including cell viability and mitochondrial activity. Cell viability was assessed by detecting the release of lactate dehydrogenase (LDH). The results were cell viability compared to the negative control (100%). Triton X-100 was used as a positive control. As shown in Figure 1A, CNMs applied at 0.0125, 0.025, 0.05, 0.1 and 0.2% posed no effects on cell viability, but 0.4% significantly reduced the viability of IPEC-J2 cells compared with the control group and other concentrations of CNM treatment groups. Notably, compared to the control group, CNMs applied at the 0.05, 0.1 and 0.2% levels significantly increased cell viability. The results show that CNMs did not cause cytotoxicity to IPEC-J2 even though they were 4 × MIC. To more deeply assess whether CNMs had an effect on mitochondrial activity in IPEC-J2 cells, we conducted an MTT assay (Figure 1B). Consistent with the LDH results, IPEC-J2 cells treated at 0.0125, 0.025, 0.05, 0.1 and 0.2% graduated concentrations of CNMs did not reduce the metabolic activity of IPEC-J2 cells. Compared with the control group, the 0.05, 0.1 and 0.2% levels of CNMs sharply increased the cell mitochondrial activity of IPEC-J2 cells, even at 0.2%, equivalent to 4 × MIC (Figure 1B). However, CNMs at the 8 × MIC level remarkably decreased mitochondrial activity moderately compared with the other treatment groups (Figure 1B). Importantly, CNMs treatment did not cause toxicity to epithelial cells even at higher concentrations than the MIC level.

### 2.2. Cytotoxicity of Safe Concentrations of CNMs on IPEC-J2 Cells Treated for Different Times

**Figure 2:**
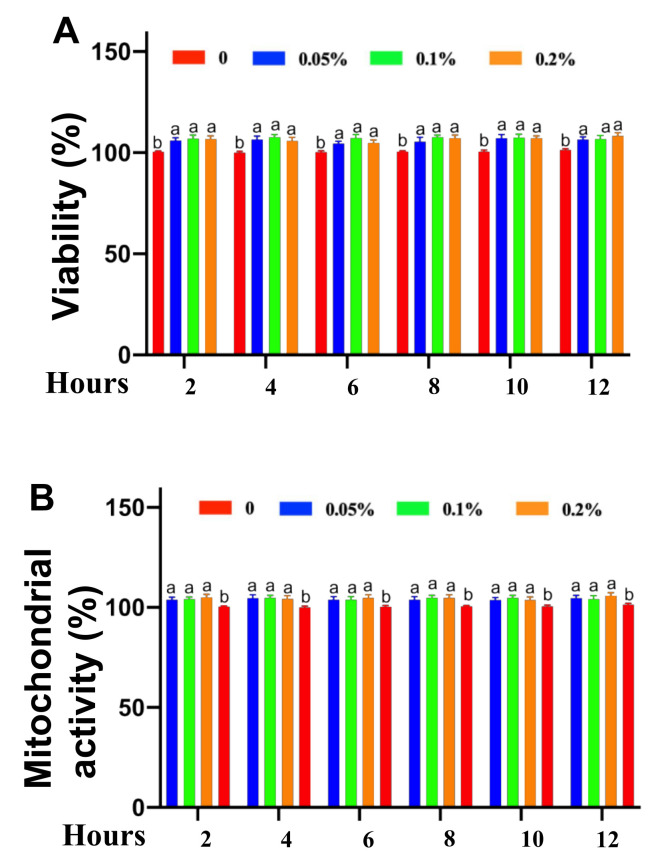
Cytotoxicity analysis of safe concentrations of CNMs toward intestinal epithelial cells at different times. LDH assay (**A**) and MTT assay (**B**) of IPEC-J2 cells treated at various concentrations of CNMs for different times. Data are means ± SEM from 6 biological data. Means with different lowercase letters differ significantly (*p* < 0.05).

Based on the above results, IPEC-J2 cells were cultured with 0.05, 0.1 and 0.2% CNMs or without CNMs for different times, such as 2, 4, 6, 8, 10 and 24 h. Figure 2 shows the cell viability and mitochondrial activity of IPEC-J2 cells at different times under different concentrations of CNM treatment. Cell proliferation was quantified by the percentage of cells in the control group. At 2, 4 6, 8, 10 and 12 h, 0.05, 0.1 and 0.2% CNMs increased IPEC-J2 cell viability (Figure 2A) and mitochondrial activity (Figure 2B) compared with the 0% CNM group. No differences in cell viability (Figure 2A) or mitochondrial activity (Figure 2B) were detected among the 0.05, 0.1 and 0.2% CNM groups at different treatment times.

### 2.3. Killing Curve and Antimicrobial Activity of CNMs

**Figure 3:**
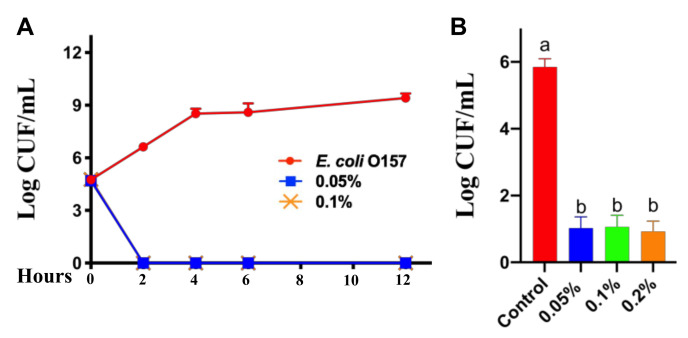
CNMs effectively inhibited *E. coli O157* growth and adhesion to IPEC-J2 cells. (**A**) Bactericidal curves of CNMs against *E. coli O157* in LB broth medium containing different concentrations of CNMs. (**B**) Monolayer IPEC-J2 cells were incubated with various levels of CNMs with or without *E. coli O157*. The adhesion of *E. coli O157* in IPEC-J2 cells was tested. Data are the means ± SEM of three independent experiments. Means with different lowercase letters differ significantly (*p* < 0.05).

Initially, we launched a time-course antimicrobial assay. The survival curve of pathogenic *E. coli O157* in the presence of CNMs was determined. A range of CNM concentrations with *E. coli O157* of stationary phase were cultured and then *E. coli O157* was collected at 2, 4, 6, 8 and 12 h to count viable cells. As shown in Figure 3A, CNMs completely eliminated *E. coli O157* after 2 h of incubation when the final concentrations reached 0.05, 0.1 and 0.2%. Notably, no bacterial regeneration was observed when the incubation time was extended to 12 h (Figure 3A). Thus, the survival curves based on log reduction data indicate that CNMs did exhibit strong antibacterial activity.

To further clarify the antibacterial activity of CNMs, an in vitro bacterial adhesion assay was performed. IPEC-J2 cells were treated with CNMs for 2 h and kept in culture medium during *E. coli O157* infection. As shown in Figure 3B, based on log reduction data, the CNM groups at concentrations of 0.05 and 0.1% significantly reduced the *E. coli O157* counts (Figure 3B). However, the results show no significant difference between the antimicrobial properties of CNMs.

### 2.4. CNMs Effectively Inhibited E. coli O157-Induced Cellular Damage

**Figure 4:**
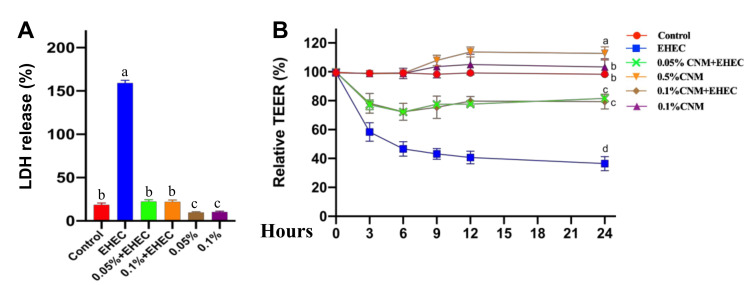
CNMs ameliorated *E. coli O157*-induced epithelial function damage in IPEC-J2 cells. Monolayer IPEC-J2 cells were cultured with or without CNMs in the absence or presence of *E. coli O157*. LDH release (**A**) and TEER (**B**) were determined. Data are the means ± SEM of three independent experiments. Means with different lowercase letters differ significantly (*p* < 0.05).

On the basis of the strong antimicrobial activity and beneficial effects of CNMs, we first examined the effects of CNMs on *E. coli O157*-induced IPEC-J2 cell membrane disruption. Intestinal permeability and transepithelial electrical resistance (TEER) were evaluated. LDH release assays showed that 0.05 and 0.1 CNMs treatments significantly reduced LDH release into the medium compared to the control group (Figure 4A). As expected, the amount of LDH in *E. coli O157*-treated IPEC-J2 cell cultures precultured with 0.05 and 0.1 CNMs was significantly decreased compared with that in the *E. coli O157* treatment group, suggesting that LDH improved the epithelial barrier of IPEC-J2 cells. However, the differences between CNMs treatment groups were not statistically significant.

To further demonstrate the beneficial role of CNMs in improving intestinal epithelial barrier function, TEER was measured at 3, 6, 9, 12 and 24 h to evaluate the integrity of tight junctions. IPEC-J2 cells were cocultured with 0, 0.05 and 0.1% CNMs. TEER in IPEC-J2 cells was calculated as the percentage of the value at 0 h. Compared with the control treatment group, 0.05% of CNMs significantly increased TEER at 12 and 24 h, but 0.1% of CNMs had no great effects on TEER. Importantly, pretreatment with 0.05 and 0.1% CNMs decreased the *E. coli O157*-induced TEER reduction compared to the *E. coli O157* treatment group (Figure 4B). These findings indicate that CNMs could have functions on the epithelial physical barrier.

### 2.5. CNMs Improved Occludin Expression and Inhibited TNF-α Expression

**Figure 5:**
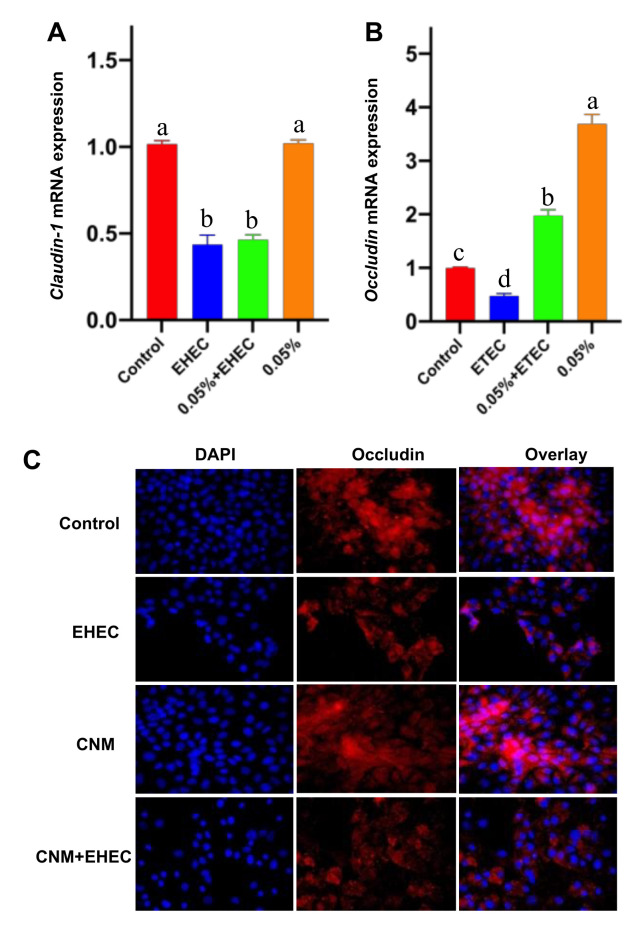
Effects of CNMs on the expression of tight junction proteins. *Claudin-1* (**A**) and *occludin* (**B**) mRNA expression after *E. coli O157* infection was measured by real-time PCR. (**C**) Occludin visualization expression (shown in red) and its combination with DAPI to visualize the nuclei (shown in blue). Data are means ± SEM. Means with different lowercase letters differ significantly (*p* < 0.05).

Tight junction proteins (TJPs) are critical for the gut barrier to defend against exogenous substances [29,30]. To further clarify the protective capacity of CNMs on the *E. coli O157*-induced disruption of TJPs, for example, the expression of *claudin-1* and *ocludin* was detected by real-time PCR and Western blotting. As shown in Figure 5A, compared with the control group, *claudin-1* and *occludin* mRNA expression was significantly decreased by *E. coli O157* treatment. However, *occludin* mRNA expression was significantly increased in CNM-pretreated IPEC-J2 cells in the *E. coli O157* infection group. Compared with the control group, the relative mRNA expression of *claudin-1* was not affected by CNMs (Figure 5B). Moreover, pretreatment of CNMs with IPEC-J2 cells did not inhibit the decrease in *claudin-1* mRNA expression caused by *E. coli O157* infection (Figure 5B).

Furthermore, confocal immunohistochemistry was applied to further confirm the protein expression of occludin (Figure 5C), where the expected colocalization of occludin protein was observed. In line with the Western blot analysis, compared with the control treatment, *E. coli O157* infection caused a decrease in the distribution of occludin, whereas CNM pretreatment was able to protect cells and enhance the distribution of occludin after *E. coli O157* challenge.

### 2.6. CNMs Relieved Inflammatory Responses by Decreasing Proinflammatory Cytokine Levels

**Figure 6:**
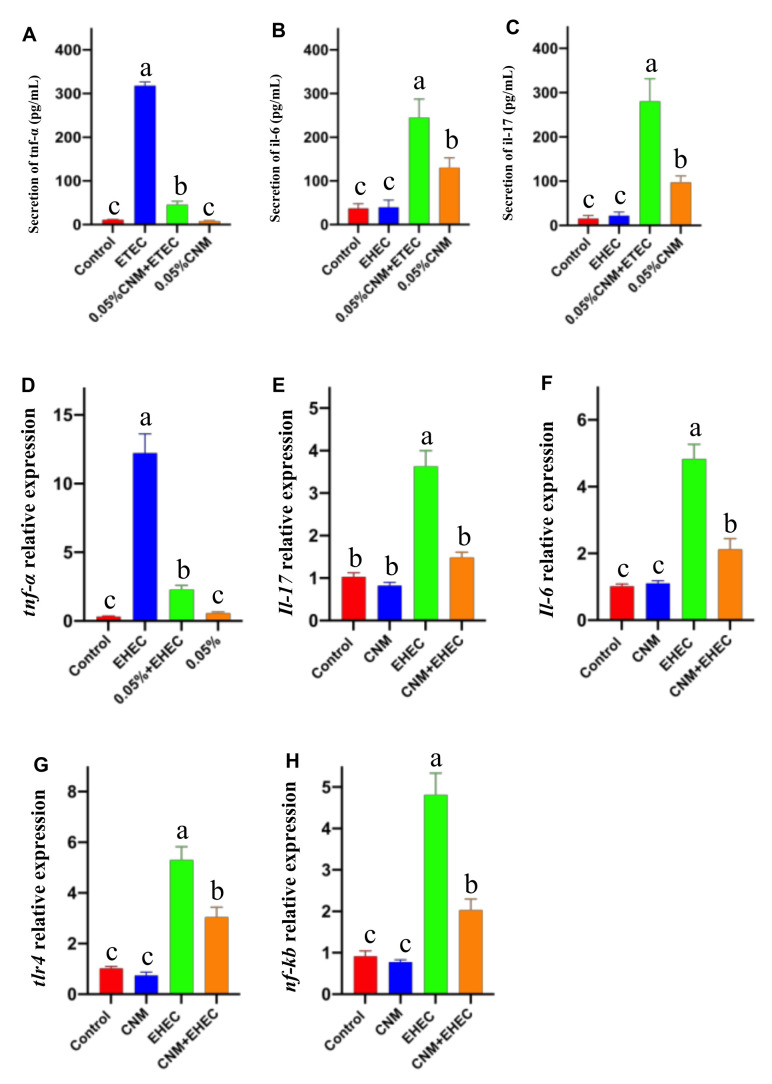
CNMs significantly reduced proinflammatory cytokine production and mRNA expression in *E. coli O157*-infected IPEC-J2 cells. Cell supernatant and cells were collected and proinflammatory cytokine production (**A**–**C**) and mRNA expression (**D**–**H**) were analyzed. Data are means ± SEM from 6 biological data. Means with different lowercase letters differ significantly by Tukey’s test (*p* < 0.05).

*E. coli O157* causes intestinal inflammation [26,27]. Therefore, the second step was to evaluate whether CNMs possessed potent anti-inflammatory effects and we measured and compared the levels of TNF-α, IL-6 and IL-17. As shown in Figure 6, the concentrations of TNF-α, IL-6 and IL-17 in the culture supernatant were higher than those in the other treatment groups after 3 h of *E. coli O157* treatment of IPEC-J2 cells. The levels of TNF-α (Figure 6A), IL-6 (Figure 6B) and IL-17 (Figure 6C) were significantly reduced by CNMs pretreatment prior to exposure to *E. coli O157*. In addition, the secretion of TNF-α, IL-6 and IL-17 was significantly lower in the CNMs-treated group than in the control group. Consistent with the secretion of the proinflammatory cytokines TNF-α, IL-6 and IL-17, *E. coli O157* induced an increase in *TNF-α*, *IL-6* and *IL-17* mRNA expression in IPEC-J2 cells compared with the control (Figure 6D–F), whereas CNMs significantly inhibited the *E. coli O157*-induced increase in *TNF-α*, *TLR4* and *NF-kB* gene expression. There was no significant difference between the CNMs control and treatment groups (Figure 6G,H).

**Figure 7:**
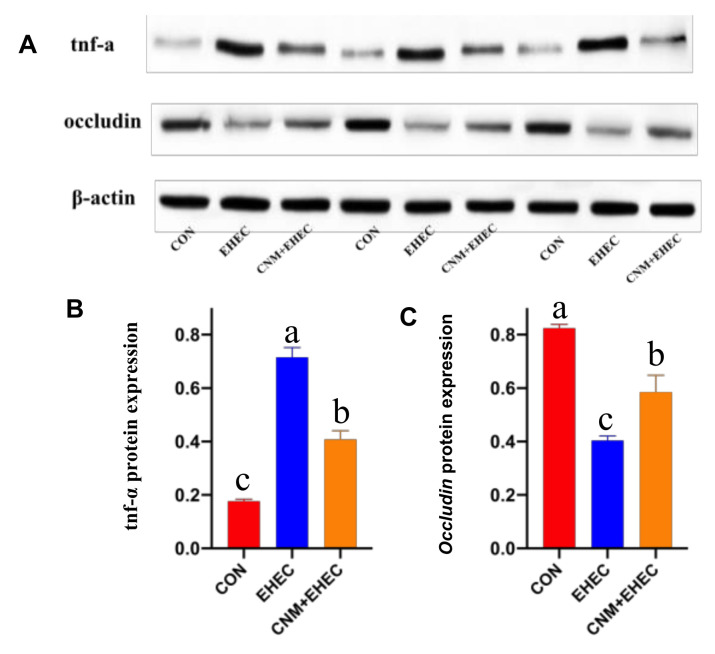
CNMs effectively decreased TNF-α and occludin protein expression in *E. coli O157* infection. IPEC-J2 cells were treated with or without CNMs and then treated with *E. coli O157*. Cells were collected and the relative abundance of TNF-α (**A**, **B**) and occludin (**A**, **C**) were measured by Western blot. Data are means ± SEM, n = 3. Means with different lowercase letters differ significantly by Tukey’s test (*p* < 0.05).

Furthermore, we performed a Western blotting assay to further identify whether pretreatment with CNMs can improve the expression of occludin and decrease TNF-α protein abundance in *E. coli O157*-treated IPEC-J2 cells (Figure 7). In agreement with the mRNA expression data, IPEC-J2 cells challenged with *E. coli O157* showed significantly decreased occludin protein expression (Figure 7A) and increased TNF-α protein expression (Figure 7B) compared to those in all treatment groups. However, pretreatment with CNMs significantly inhibited the *E. coli O157*-induced downregulation of occludin protein expression and increase in TNF-α protein expression.

### 2.7. CNMs Attenuated Proinflammation by Inhibiting Mitogen-Activated Protein Kinase and Nuclear Factor Κb Pathway Activation

**Figure 8:**
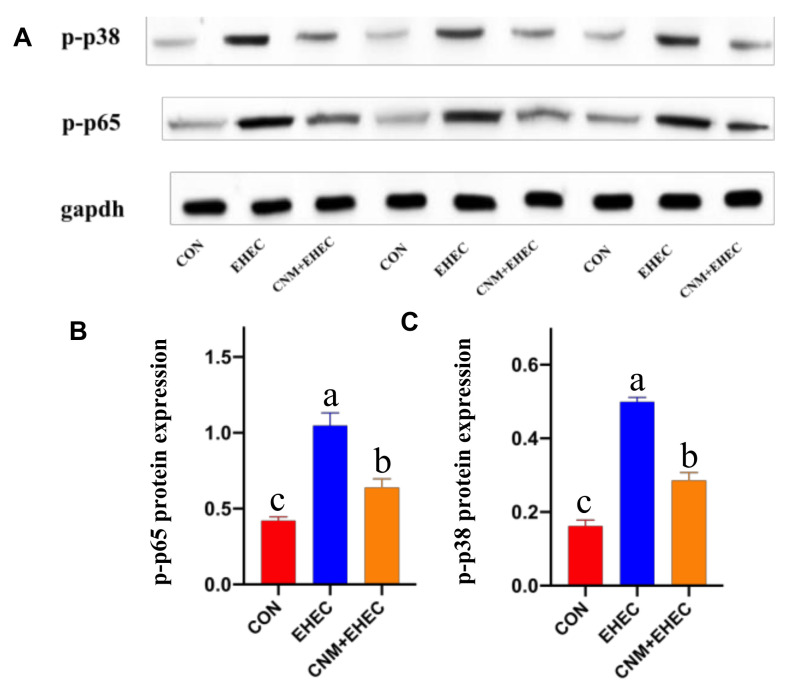
Phosphorylated NF-κB and p38 MAPK pathways were analyzed by Western blotting. Cells were collected after *E. coli O157* challenge. (**A**) Presentive image of the bands of phosphorylated p38 and phosphorylated NF-Κb. (**B**) Relative protein abundance of *p*-NF-κB and (**C**) *p*-p38. The results are presented as means ± SEM. n = 3. Means with different lowercase letters differ significantly by Tukey’s test (*p* < 0.05).

Based on the above experiments, the data indicate that CNMs inhibited inflammatory responses. The NF-κB and MAPK pathways are key cellular cascade responses involved in the inflammatory response [7,28]. As shown in Figure 8, phosphorylated NF-κB (Figure 8A) and P38 (Figure 8B) protein abundance was significantly higher in the *E. coli O157*-infected group than in all treatment groups. However, the abundance of phosphorylated NF-κB protein was significantly lower in CNM-treated IPEC-J2 cells than in the *E. coli O157*-treated group. The key point is that there was no significant difference between the CNMs pretreatment group and the control group. This result illustrates that CNMs could inhibit the activation of the NF-κB and MAPK pathways, thus achieving relief from inflammation.

### 2.8. CNMs Improved Cell Damage and Inflammatory Responses by Attenuating Oxidative Stress Caused by E. coli O157 Infection

**Figure 9:**
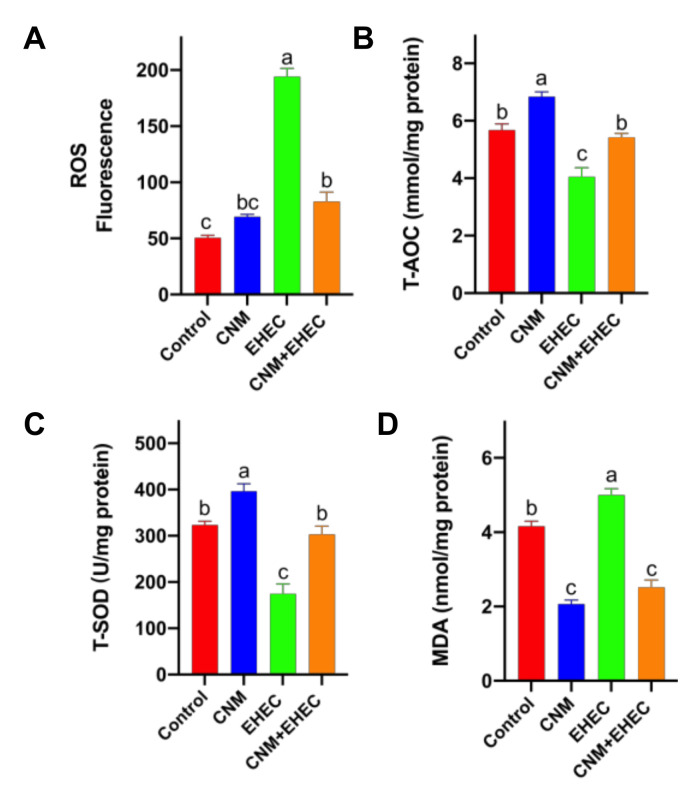
Effects of CNM treatment on oxidative stress induced by *E. coli O157* infection in IPEC-J2. Cells were cultured with or without CNMs in the presence or absence of *E. coli O157*. (**A**) Quantitative assay of ROS in IPEC-J2 cells; (**B**) T-AOC; (**C**) T-SOD; (**D**) MDA content. These enzymes were detected with commercial reagent kits. Data are given as means ± SEM (n = 6). Means with different lowercase letters differ significantly by Tukey’s test (*p* < 0.05).

In addition, oxidative stress inflammation has become a major concern in the food, medical and agriculture industry applications [31,32]. To examine whether CNMs at working concentrations could inhibit foodborne pathogen-induced oxidative stress, reactive oxygen species (ROS) and antioxidant enzyme activities were determined in intestinal epithelial cells (Figure 9). The results show that CNMs did not induce a significant amount of ROS generation when IPEC-J2 cells were incubated with CNMs alone. The fluorescence in CNMs treated with IPEC-J2 cells did not differ from that in the controls (Figure 9A). However, *E. coli O157* treatments caused the fluorescence to be significantly higher than that of the control and CNMs-only treatments. Although pretreatment with CNMs had a higher fluorescence compared with the control, pretreatment with CNMs significantly decreased the ROS generation caused by *E. coli O157* and the amount of ROS was not significantly different compared with CNMs alone.

In the antioxidant enzyme activity assay, compared with the control, CNMs alone remarkably increased the activities of total antioxidant capacity (T-AOC) (Figure 9B) and superoxide dismutase (SOD) activities (Figure 9C) and significantly decreased the level of malondialdehyde (MDA) (Figure 9D), whereas *E. coli O157* infection caused an increase in MDA levels and a decrease in T-AOC and SOD activities. Notably, pretreatment with CNMs resulted in lower MDA levels than *E. coli O157* and the control treatments and the level of MDA was similar to that of CNMs alone. The T-AOC and SOD activities in the pretreatment CNMs group were significantly higher than those in the *E. coli O157* group and were similar to those in the control group.

## 3. Discussion

The history of human development is, to a certain extent, a history of constant struggle against various diseases and bacterial infections in different domains exhibit mounting public health threats [1,2,3,33]. In this paper, we evaluated the potential protective capacity of CNMs, a novel antimicrobial engineered with antimicrobial peptides and chitosan nanoparticles, against foodborne pathogenic *E. coli O157* infection in an IPEC-J2 cell model. Our study demonstrates that the CNMs did not cause cytotoxicity to the IPEC-J2 cell line, while they exhibited excellent bactericidal activity against *E. coli O157* and significantly decreased bacterial adhesion in cells. Genotoxicity was tested through the ROS assay, which indicated that CNMs at working levels did not increase ROS generation. Moreover, CNMs could effectively improve intestinal barrier dysfunction and alleviate inflammation caused by *E. coli O157*. Moreover, CNMs improved cell damage and inflammatory responses by attenuating oxidative stress caused by *E. coli O157* infection.

Clinically, antibiotics play an important role in the treatment of most infectious diseases, but the increase in resistance and emergence of multidrug-resistant pathogens currently poses a serious health risk to humans and animals [33,34,35,36]. Additionally, a large number of food spoilage problems and pathogenic bacteria has been shown to be present in various food matrices, all of which can lead to infection in humans and animals [8,37]. After that, the spread of resistant strains from food to humans or human food animals treated with antibiotics caused a further increase in antibiotic resistance. As a result, antibiotics are becoming less effective in treating various bacterial infections as bacterial resistance grows more severe [38,39]. If the problem of bacterial resistance is not addressed in a timely manner, we will regress back to the pre-antibiotic era. Therefore, it is urgent to seek effective solutions to address the problem of antibiotic resistance. Nanomaterials have shown great potential in the treatment of severe drug-resistant bacterial infections due to their excellent size effect, specific physicochemical properties and ease of modification.

Microcins and chitosan have now been defined as promising antimicrobial compounds or drug delivery with a wide scope and potential in the food, health and veterinary sectors [17,18,19,20,21,22,23]. Among our previous results, we successfully designed nano CNMs, which are alternative antimicrobial agents with development potential. It has great advantages and potential as a novel antimicrobial agent against multidrug pathogenic infections in the food and animal industry [24]. However, experience with the use of polymers as antimicrobial agents is still scarce for the food and animal industries and further studies are needed to ensure their safety and efficacy before they can be widely used. For example, it is of great interest to clarify the mechanisms by which CNMs act at the cellular level to avoid undesirable side effects. In addition, foodborne pathogenic *E. coli O157* can cause intestinal diseases in humans and livestock, destroy the intestinal barrier, aggravate systemic inflammation and seriously threaten human health and livestock development [5,6,7,40].

To address the above-presented issues, based on the previous minimum inhibitory concentration (MIC) data of CNMs against *E. coli O157* [24], we evaluated the bactericidal activity and the results showed that CNMs had powerful killing activity against *E. coli O157*. Compared with MccJ25 and CNs/CMs alone [22,24], it showed the same strong bactericidal activity against *E. coli*. The cytotoxicity of mammalian cells is an important limiting factor in the application of antimicrobial agents [17,41,42]. The results of this study showed that, although CNMs have strong antibacterial activity, they do not cause IPEC-J2 cytotoxicity, but they lead to cell membrane damage and reduced cellular activity when the concentration increases, suggesting that there are potential risks for nano-antimicrobial polymers, significantly limiting their further applications.

A prerequisite for widespread use is the need to ensure the safety and effectiveness of CNMs. This paper addresses the effects of CNMs on *E. coli O157*-induced intestinal inflammation and barrier dysfunction because CNMs significantly reduce pathogens on the surface of IPEC-J2 cells and improve cell viability. Based on the results of cytotoxicity experiments, we chose 0.05% and 0.1% CNMs to examine their effects on subsequent experiments. First, LDH and TEER are typical indicators of epithelial integrity. Impaired mucosal barrier function can enhance the release of LDH from cell culture media and TEER is a measure of intestinal epithelial permeability [28,43]. We found that CNMs significantly reduced the increase in LDH release and decrease in TEER in *E. coli O157*-induced epithelial cells. These findings suggest that CNMs may form a physical barrier between pathogens and enterocytes or induce intestinal epithelial cells with the ability to resist infection. Notably, CNMs mediate intestinal barrier function.

The main types of intestinal barriers are the intestinal epithelial barrier, the immune barrier, the chemical barrier and the biological barrier. The intestinal epithelial barrier is the first barrier that prevents toxic and harmful substances such as bacteria and antigens from entering the lower intestinal mucosa and the blood. Tight junction (TJ) structures mainly include TJPs by zo-1, ocludin, claudin, etc., which are a key part of the intestinal epithelial barrier system and occupy an important position in intestinal epithelial cells [44,45,46,47]. Next, to further test the effects of CNMs on tight junctions, we measured the expression of TJPs. Importantly, CNMs significantly increased the mRNA and protein abundance of ocludin in *E. coli O157*-treated cells and occludin protein is one of the critical connexins [48]. Notably, unlike the control group, the CNMs-treated group did not significantly affect the mRNA expression of claudin-1, which is not surprising, as this difference could be a possible change in the mode of action. Thus, these results tentatively suggest that CNMs could protect the integrity of the intestinal epithelium by directly killing pathogens and forming a physical barrier between pathogens and intestinal cells, as well as potentially inducing the ability of intestinal cells to resist infection. However, there is still much work for improvement. For example, understanding the mechanism by which CNMs function in intestinal barrier integrity.

Chronic excess production of proinflammatory cytokines can lead to intestinal damage and high energy demands. There is an association between epithelial barrier damage and immune-mediated diseases [44,49,50]. Our findings are essentially the same as those of previous studies, in which *E. coli* induced the production and expression of proinflammatory cytokines in epithelial cells, possibly regulating cellular barrier function and constructing an inflammatory environment around the epithelial barrier. In this paper, the secretion and expression of *E. coli O157*-induced proinflammatory cytokines such as TNF-α, TLR4 and NF-κB were inhibited by pretreatment with CNMs. The regulation of proinflammatory cytokine expression is influenced by the activation of MAPK and NF-κB signaling pathways. Numerous studies have demonstrated the effects of TNF-α, IL-6 and IL-8 on intestinal permeability, presumably because NF-κB and MAPK signaling molecules play a role in proinflammatory cytokine expression, such as activation of the p38 MAPK and NF-κB pathways, and promote TNF-α production and gene expression in vitro and in vivo [45,51,52]. The results of this paper demonstrate the mechanism of action of CNMs, which may significantly inhibit the expression of foodborne pathogenic *E. coli O157*-triggered inflammatory factors through downregulation of the MAPK and NF-κB pathways. In addition, some previous studies have shown that there is a correlation between MAPK activation and barrier dysfunction, which may be mainly mediated or linked by proinflammatory cytokines. TJ regulation (repair, assembly and disassembly) has been proposed according to different physiological and pathological conditions [47,49]. In future studies, in-depth analysis of other signaling pathways is needed to clarify other pathways of CNMs in TJ regulation and anti-inflammatory responses in vivo.

Previous studies have shown that oxidative stress can be caused by nanoparticles and this issue is becoming a major concern when nano-antimicrobial polymers are applied in the food, health and medical industries [53,54,55]. In the body, oxidants and antioxidants are always balanced. Once the balance is disrupted, the oxidative stress response occurs rapidly and a large amount of ROS is produced, accompanied by excessive production of hydroxyl free radicals and oxygen free radicals. These excess ROS and free radicals can lead to an increase in proinflammatory cytokines, destruction of redox homeostasis, cell death and damage to intestinal mucosal structure or function, finally resulting in severe intestinal inflammatory disease and increased mortality [56,57]. Additionally, the activation of the p38 MAPK signaling pathway can reduce the production of ROS, thereby alleviating lipopolysaccharide (LPS)-induced inflammatory injury reactions to achieve anti-inflammatory effects, reducing the level of intracellular ROS and inhibiting LPS-induced cell injury [21,31,32].

In the present study, we found that CNMs alone did not increase ROS generation, indicating that the generation of ROS might also be a concern for using CNMs to treat pathogenic bacteria because ROS can cause an increase in the mutation rate of bacteria, resulting in multidrug resistance [58]. Furthermore, pretreatment with CNMs significantly decreased the ROS generation caused by *E. coli O157*; critically, the amount of ROS was not significantly different compared to CNMs alone. The results indicate that CNMs exerted antioxidant ability to decrease the ROS caused by *E. coli O157* infection, suggesting that CNMs could relieve the inflammatory response by inhibiting oxidative stress by reducing ROS production. On the other hand, CNMs did not induce genotoxicity. This result is consistent with a CN study. Ma et al. demonstrated that CNs did not cause genotoxicity without increasing ROS generation.

Endogenous antioxidant enzymes such as SOD and T-AOC are the main defense strategies to resist the oxidative damage caused by ROS and maintain the redox balance of cells. Immune function can be enhanced by increasing antioxidant enzyme activities and decreasing MDA levels, eliminating excessive ROS in the body and, ultimately, promoting health [59,60]. In this study, our results show that pretreatment with CNMs resulted in lower MDA levels and higher T-AOC and SOD activities than *E. coli O157* infection and were similar to the control group, indicating that CNMs could relieve oxidative stress by increasing antioxidant enzyme activities to eliminate ROS and decreasing inflammatory responses.

## 4. Materials and Methods

### 4.1. Preparation of Chitosan Nanoparticles

The CNs were prepared according to a previously developed protocol with slight modifications [21,22]. In short, 2% chitosan (*w/v*; 448869, Sigma-Aldrich, St. Louis, MO, USA) was dissolved in 2% acetic acid (*v/v*; Thermo Fisher Scientific Inc., Waltham, MA, USA) and mixed with 1% Tween 80 (*v/v*; Acros Organics, Morris, NJ, USA). Then, 10% sodium sulfate (*w/v*; Thermo Fisher Scientific Inc., Waltham, MA, USA) solution was added dropwise to the chitosan solution for ultrasonic crosslinking. Ultrasonication was continued for 25 min and CNs were collected by centrifugation at 14,000 rpm. CNs were washed three times with sterile Milli-Q water and stored at 4 °C before use.

### 4.2. Bioconjugation of Microcin J25 to CNs

MccJ25 containing 21 amino acids was provided by our research team, Prof. Qiao’s lab. The peptide was prepared as previously described [28]; the sequence and molecular weight were GGAGHVPEYFVGIGTPISFYG and 2107 Da. Purified MccJ25 was 99.96%. The conjugation of CNs–MccJ25 (CNMs) was performed as follows: CNs were dissolved in 0.1 M sodium acetate (Thermo Fisher Scientific Inc., Waltham, MA, USA) buffer (pH 5.5) to 1% (*w/v*). MccJ25 was added to the solution (CN:MccJ25 ratio = 10:1, *w/w*). Then, 1-ethyl-3-(3-dimethyl aminopropyl) carbodiimide (EDC; 0.5 mM; Sigma-Aldrich, St. Louis, MO, USA) and sulfo-N-hydroxysulfosuccinimide (sulfo-NHS; 0.25 mM; Sigma-Aldrich, St. Louis, MO, USA) were immediately added to the mixture, stirred at 4 °C and left overnight. Conjugators, CNMs, were permeabilized by Mill-Q water (dialysis membrane molecular weight cutoff 12–14 kDa). Equal volumes of water were replaced every 1 h until 24 h. After dialysis, CNMs were freeze-dried overnight. CNMs lyophilized powder was dissolved in sterile Mill-Q water and stored at 4 °C until assayed [24].

### 4.3. Cell and Culture Conditions

Intestinal porcine epithelial cell J2 cell lines were used in this study. Cells were cultured in Dulbecco’s modified Eagle medium F12 (DMEM/F12; Corning Incorporated, Corning, NY, USA) supplemented with 20% fetal bovine serum (FBS; HyClone, Logan, UT, USA). Cells were inoculated in 24- or 96-well plates and incubated for 24 h prior to CNMs treatment.

### 4.4. Cytotoxicity Analysis

The LDH cytotoxicity (Clontech, Mountain View, CA, USA) assay kit was used to determine the amount of LDH released and to detect whether CNMs caused cell membrane damage. IPEC-J2 cells (2 × 10^4^) were individually inoculated in 96-well plates and cultured to 80–90% confluence. CNMs (0.0125, 0.025, 0.05, 0.1, 0.2% and 0.4%) were added to the cells and incubated for 24 h. We set 1% Triton X-100 as a positive control. One hundred microliters of cell culture medium per well was transferred to a new well. The catalyst and dye solution were mixed and added to the medium and incubated for 30 min. The absorbance was measured at 490 nm and 670 nm using a fluorescence microplate reader (SynergyMx, BioTek, Winooski, VT, USA). The absorbance value was corrected by subtracting the reading at 670 nm from the reading at 490 nm. The absorbance values of 1% Triton X-100 dissolved in medium treatment (100% cell death) and negative control (0% cell death) were normalized. This assay was performed three times.

An MTT Cytotoxicity Assay Kit (Life Technologies Corporation, Eugene, OR, USA) was used to detect whether CNMs affected cellular metabolic activity. A total of 2 × 10^4^ cells were added to each of the 96-well plates and incubated for 24 h. Then, CNMs, at the same concentration used for the LDH assay, were added to the cells and incubated for 24 h. We set 1% Triton X-100 as a positive control. Each well was spiked with 20 μL of MTT solution (5 mg/mL in Dulbecco phosphate-buffered saline, DPBS) and incubated for 3.5 h at 37 °C and 5% CO_2_. MTT metabolites were collected by centrifugation and then resuspended in 200 μL of dimethyl sulfoxide. The optical density was read at 560 nm and the background was subtracted at 670 nm. The absorbance values of 1% Triton X-100 treatment (100% cell death) and negative control (0% cell death) were normalized.

Based on the above results, IPEC-J2 cells were cultured with different levels of CNMs or without CNMs for different times, such as 2, 4, 6, 8, 10 and 12 h. Cell viability and mitochondrial activity of IPEC-J2 cells were tested.

### 4.5. Antimicrobial Activity Curves

The second step was to generate the antimicrobial activity profile of the CNMs with the best activity. Samples of *E. coli O157:H7* were prepared in Luria–Bertani (LB) broth with final CNMs concentrations of 0%, 0.05% and 0.1%. Inoculated samples were incubated at 37 °C with continuous agitation (200 rpm). Then, mixtures were plated every 2 h for 12 h on LB agar Petri dishes and, after 24 h, complete elimination of *E. coli O157:H7* was verified. LB agar plates were incubated overnight at 37 °C and bacterial counts were obtained from three independent trials.

### 4.6. Adherence Assay

The adhesion assay was used to evaluate the adhesion of representative strains to IPEC-J2 cells. IPEC-J2 cells were preserved in DMEM/F12 (10-017-CV; Corning, NY, USA) supplemented with 20% (*v/v*) FBS at 37 °C and 5% CO_2_. Approximately 10^5^ IPEC-J2 cells were inoculated into each well of a 6-well polystyrene plate and then incubated until IPEC-J2 cells were 85–90% fused. Prior to infection, bacterial cells were incubated for 2 h with medium alone or medium containing CNMs. Overnight cultures of *E. coli O157* in LB broth were seeded in new LB broth (1:250) tubes to obtain a main culture and incubated for 8 h at 37 °C in shaking flasks. The 106 bacterial cells were washed 3 times with sterile PBS and resuspended in 500 µL DMEM with 10^5^ IPEC-J2 cells per well (MOI 1:10). After incubation at 37 °C with 5% CO_2_ for 3 h, the medium was replaced with 500 µL of fresh DMEM per well and then incubated for another 3 h. After removing the medium, each well was rinsed 3 times with sterile PBS to remove unattached bacteria. Then, 1 mL of 1% Triton X 100 was added to each well to lyse IPEC-J2 cells. Finally, 100 µL of the diluted suspension was applied to LB agar and incubated overnight at 37 °C; then, colonies were counted on the plates. This experiment was repeated three times.

### 4.7. Measurement of TEER and LDH

IPEC-J2 cells (2 × 10^5^) were implanted into 6-well transwell PTFE filters (pore size, 0.4 µm; 4.7 cm^2^; Costar, Corning Inc., Corning, NY, USA) and grown to confluence. After sample isolation, the cells were treated. Cells were divided into four groups: control (untreated), *E. coli O157* (final concentration 2.5 × 10^6^ CFU/mL *E. coli O157* treatment for 3 h), CNMs (0.05 and 0.1% CNMs treatment for 24 h) and CNMs + *E. coli O157* (addition of 0.05 and 0.1% CNMs treatment for 24 h, final concentration 2.5 × 10^6^ CFU/mL *E. coli O157* treatment for 3 h) to determine LDH activity.

To more deeply investigate the protective effect of *E. coli O157* CNMs induced by membrane damage, cells differentiated from IPEC-J2 were used with or without 0.05 and 0.1% CNMs indexing for 3, 6, 9 and 12 h in the presence or absence of *E. coli O157* for 3 h. TEER was measured.

### 4.8. Determination of Proinflammatory Cytokines

The levels of cytokines (TNF-α, IL-6 and IL-17) were assessed by adding *E. coli O157* to IPEC-J2 cells in DMEM/F12 with or without 0.05% CNMs added to serum (without antibiotics). The secretion of cytokines in culture supernatants was measured using an enzyme-linked immunosorbent assay kit (ELISA) purchased from Nanjing Jiancheng Biology Engineering (Nanjing, China). Concentrations were quantified based on absorbance at 450 nm on an enzyme marker (Bio-Rad Laboratories, Hercules, CA, USA).

### 4.9. Real-Time PCR for mRNA Expression Analysis

Cells were lysed directly in TRIzol (Invitrogen, Carlsbad, CA, USA). Total RNA was extracted according to the manufacturer’s instructions. One microgram of total RNA was synthesized using the PrimeScript First Strand cDNA Synthesis Kit (Takara, Dalian, China) and reverse-transcribed according to the manufacturer’s protocol; first strand cDNA was synthesized and stored at −80 °C. Real-time PCR was performed on an Applied Biosystems 7500 real-time PCR system (Applied Biosystems, Foster city, CA, USA) using the SYBR Green PCR Master Mix (Takara, Dalian, China) as previously described [26,30]. β-actin was used as an endogenous control. The primers used are shown in Table 1.

### 4.10. Western Blot and Immunofluorescence Analysis

Cells were collected based on Western blotting as described previously and analyzed for protein abundance [26,30]. Membranes were incubated with primary antibodies (ocludin, tnf-α, *p*-P38 and NF-κB (*p*-p65) (Santa Cruz Biotechnology, Santa Cruz, CA, USA) at 4 °C overnight and then washed. The membranes were then incubated with horseradish peroxidase (HRP)-labeled secondary antibody (Applygen Technology, Inc., Beijing, China) for 1 h at room temperature. Signal detection was performed using the ImageQuant LAS 4000 microsystem (GE Healthcare Biosciences AB, Inc., Uppsala, Sweden), Western Blot brightness reagents (Applygen, Beijing, China) and ImageQuant TL software (GE Healthcare Life Science, Uppsala, Sweden) for gel imaging systems.

The expression levels of the intercellular TJP occludin were assessed by immunofluorescence microscopy. Briefly, IPEC-J2 cells were incubated with a rabbit anti-occludin antibody (Abcam, Boston, MA, USA) and then with FITC-conjugated goat anti-rabbit secondary Ab. After washing with PBS, cells were removed from the plastic holder and mounted on a glass slide that contained a DAPI Vectashield and examined with a Leica TCS SP5 confocal laser microscope (Keenes, Osaka, Japan).

### 4.11. Determination of Antioxidant Activity

IPEC-J2 cells were treated by sonication (Sonic VCX105, Sonics & Materials Inc., CT, USA) in ice-cold PBS and, for complete debris removal, they were centrifuged at 11,000 rpm for 15 min. SOD activity was measured by TMB reaction spectrophotometry (550 nm) and thiobarbituric acid reaction spectrophotometry (450 nm). T-AOC was determined based on the reaction with Fe3+ and o-phenanthroline at 520 nm. MDA levels, SOD activity and T-AOC were determined using commercially available kits (Nanjing Jiancheng Bioengineering, Nanjing, China) based on the instructions. BCA protein analysis kits (Thermo Fisher Scientific Inc. Waltham, MA, USA) were used to determine the total protein concentration.

### 4.12. ROS Assay

Reactive oxygen species production in IPEC-J2 cells treated with CNMs was measured by a fluorescent intracellular ROS kit (Sigma-Aldrich) based on the manufacturer’s instructions. The master reaction mix ROS kit (0.1 mL/well) was added to IPEC-J2 cells cultured in 96-well plates as described above and then incubated for 1 h (37 °C, 5% CO_2_). Then, CNMs were added to the wells and incubated for 24 h (37 °C, 5% CO_2_). Fluorescence intensity was measured using an enzyme marker (BioTek, Winooski, VT, USA) with λex = 530/λem = 620 nm.

### 4.13. Statistical Analysis

The results are presented as the mean ± standard error of the mean (SEM). A one-way ANOVA, followed by analysis of the data, was performed using GraphPad Prism 8 software (GraphPad software Inc., San Diego, CA, USA). Tukey’s multiple comparison test was used to determine the differences between treatments. All data were visualized using GraphPad Prism 8 version. Differences were statistically significant when *p* < 0.05.

## 5. Conclusions

Overall, our results suggest that CNMs exhibited strong bactericidal activity against foodborne pathogenic *E. coli O157* and did not induce cytotoxicity and genotoxicity in IPEC-J2 cells. In addition, CNMs pretreatment of intestinal cells was able to prevent foodborne pathogen *E. coli O157*-induced intestinal barrier function damage and oxidative stress, thereby alleviating the inflammatory situation. These findings demonstrate that CNMs at working concentrations may be applied in food and veterinary medicine. Even though such in vitro measures are limited in several ways, the ability to design better animal models (e.g., mice or pigs) based on these data will facilitate future human clinical studies. This may provide a broad scope for the application of CNMs, the treatment of infectious diseases caused by pathogens and facilitate the development of food, health and animal industries that are not dependent on antibiotics. Thus, the safety of novel nanomaterials or antimicrobials requires much attention, especially in vitro and in vivo data regarding their safety and toxicity, which are necessary before application.

## Figures and Tables

**Table 1:** Primers for gene expression using real-time PCR.

Gene	Primer Sequence	Product Size
*β-actin*	F:5’-TGCGGGACATCAAGGAGAAG-3’	216
	R: 5’-AGTTGAAGGTGGTCTCGTGG-3’	
*TNF-α*	F:5’-ATTCAGGGATGTGTGGCCTG-3’	120
	R: 5’-CCAGATGTCCCAGGTTGCAT-3’	
*IL-6*	F: 5’-TGGATAAGCTGCAGTCACAG-3’	109
	R: 5’-ATTATCCGAATGGCCCTCAG-3’	
*IL-22*	F: 5’-TCTCGGTGTAGAGCAAGG-3’	146
	R: 5’-TTCCCAAAGTGCTGGTATT-3’	
*TLR4*	F:5’-CTCCAGCTTTCCAGAACTGC-3’	192
	R: 5’-AGGTTTGTCTCAACGGCAAC-3’	
*NF-KB*	F:5’-CTCGCACAAGGAGACATGAA-3’	147
	R:5’-ACTCAGCCGGAAGGCATTAT-3’	
*IL-17*	F:5’-CAGCAAGCTCCAGCTCATCCATC-3’	92
	R:5’-CAGCAGAAGCAGCAGTGACAGG-3’	
*Claudin-1*	F: 5’-GCTGGGTTTCATCCTGGCTTCT-3’	110
	R: 5’-CCTGAGCGGTCACGATGTTGTC-3’	
*Occludin*	F: 5’-GTGGTAACTTGGAGGCGTCTTC-3’	102
	R: 5’-CCGTCGTGTAGTCTGTCTCGTA-3’	

## Data Availability

Not applicable.
